# Full-Stokes polarization imaging method based on the self-organized grating array in fused silica

**DOI:** 10.1038/s41598-018-19942-6

**Published:** 2018-02-05

**Authors:** Canhua Xu, Chaozhen Ke, Jing Ma, Yantang Huang, Zhiping Zeng

**Affiliations:** College of Physics and Information Engineering, Fuzhou University, Fuzhou, 350108 China

## Abstract

A full-Stokes polarization imaging method based on the self-organized grating array was presented. By focusing the ultra-fast laser with moderate fluence into fused silica, the self-organized grating array was fabricated, featuring the optical properties similar to wave plates. A set of four independent polarization measurements were simultaneously acquired with designed grating array mounted in the focal plane of an imaging detector. Experimental results including the device fabrication, calibration and optimization were presented. Finally, a principle verification experiment was implemented for our polarization imaging method.

## Introduction

The techniques of imaging polarimeter are capable of recording light polarization of a scene, which contains special information related to surface features, shapes, shadows, and roughness. Therefore it improves the ability of target recognition in the complex background, and also provides valuable information in environmental monitoring, remote sensing, biomedical imaging and military target detection^1–6^. After extensive research, several techniques of imaging polarimeter including division-of-time, division-of-amplitude, division-of-aperture, and division-of-focal-plane (DoFP) have been developed in the past decades^7–11^. Among them, DoFP polarimeters adopt four independent micropolarimeter on the top of neighboring pixels of the image sensor to measure the four components of a Stokes vector. This approach has significant advantages in terms of manufacturing cost, volume, weight, power dissipation and system integration. Since 1994, reactive-ion-etching (RIE) has been used to pattern dichroic polymer film and form a micropolarimeter array capable of extracting (S0, S1, S2) for partial-linear polarization imaging^12–14^. Other approaches utilize evaporated aluminum or gold film to form the birefringent micropolarizer array for the polarization imaging^15,16^. However, the birefringence of the metallic film originates from absorbing the light polarization paralleled to the grating stripe, which cannot distinguish the phase delay information of the light. The first full-Stokes DoFP polarimeter was developed in 2006^17^, which adopted subwavelength gratings in fused silica as the micropolarization elements. However, it is difficult to control the morphology and depth of the grating in fused silica by the technology of electron beam direct writing. Substantial errors exist in the experimental results. In 2010, by patterning a liquid crystal layer on top of a visible-regime metal-wire-grid polarizer, X. Zhao *et al*. demonstrated the full-Stokes measurement in principle^18^. Until 2012, the first practical full-Stokes DoFP imaging was presented in experiments through using patterned liquid crystal polymer (LCP) polarizers and retarders^19^. Nonetheless, this approach was limited in a few areas of research and applications due to the complicated fabrication of the LCP polarizers and retarders^20,21^.

On the other hand, with the rapid development of ultra-fast laser technology, the ultra-fast laser processing of inorganic non-metallic materials has become a hot research field^22,23^. Depending on the amount of deposited energy, three distinct types of modifications can be induced in the bulk of transparent materials. In particular, moderate fluences result in the spontaneous formation of gratings with sub-wavelength refractive index distribution^24–26^. The grating has the characteristics of high transmittance, rewritable and thermal stability. More importantly, it exhibits an obvious optical birefringence effect, with the slow and fast optical axes aligned, respectively, parallel and perpendicular to the grating corrugation. The period, thickness and depth of the grating can be exactly controlled by the ultra-fast laser. Therefore, both the optical axis and phase delay induced by the grating are designable^27^. Compared to the microwaveplate or micropolarizer fabricated by photolithography, the self-organized grating fabricated by ultrafast laser has the unique merit of single-step processing. By simply adjusting the writing speed of the ultrafast laser, the phase delay induced by the grating can be modified. In addition, there is no limitation to reduce the size of waveplates up to few micrometers and making them the same size as pixels in the typical CCD detector. Moreover, the nanostructure imprinted in the fused silica has excellent chemical and thermal stability. The simple processing technology, compact structure and excellent stability make the self-organized grating especially suitable for the applications in various complex environments. In this paper, we proposed a DoFP polarization imaging technique based on the self-organized grating array in fused silica. Periodical sets of four independent self-organized gratings were fabricated by the ultra-fast laser. The phase delay and the optical axis of the gratings were measured and fitted with the theory of Müller matrix. Furthermore, our setup was optimized through calculating the determinant of the Müller matrix. At last, as a proof-of-principle verification experiment, polarization imaging of a light passing through a calcium fluoride crystal was measured and discussed.

## Theory of the polarization imaging

In theory, four independent measurements are necessary for full-Stokes’ vector detection. Typically, measured light polarization S(in) is transferred to S(out) by a combination of the wave-plate and polarizer. The measurements can be expressed as Eq. (1).

1 $$[\begin{array}{c}{S}_{0}^{i}(out)\\ {S}_{1}^{i}(out)\\ {S}_{2}^{i}(out)\\ {S}_{3}^{i}(out)\end{array}]={M}^{i}({\rm{\Delta }},\theta ,\phi )[\begin{array}{c}{S}_{0}(in)\\ {S}_{1}(in)\\ {S}_{2}(in)\\ {S}_{3}(in)\end{array}]$$

Here *M* is the Müller matrix for the detection light path. Superscript *i* = 1, 2, 3, 4, represents different independent measurement. Δ, *θ* denote the phase delay and the optical axis of the wave-plate, and *φ* is the axis angle of the polarizer. Müller matrix has 4 × 4 components as follows:

2 $${M}^{i}({\rm{\Delta }},\theta ,\phi )=[\begin{array}{cccc}{M}_{11}^{i} & {M}_{12}^{i} & {M}_{13}^{i} & {M}_{14}^{i}\\ {M}_{21}^{i} & {M}_{22}^{i} & {M}_{23}^{i} & {M}_{24}^{i}\\ {M}_{31}^{i} & {M}_{32}^{i} & {M}_{33}^{i} & {M}_{34}^{i}\\ {M}_{41}^{i} & {M}_{42}^{i} & {M}_{43}^{i} & {M}_{44}^{i}\end{array}]$$

Only light intensity (i.e., S0) can be detected by the image sensor, so we rewrite the first line of Eq. (2) of four measurements as Eq. (3):

3 $$[\begin{array}{c}{S}_{0}^{1}(out)\\ {S}_{0}^{2}(out)\\ {S}_{0}^{3}(out)\\ {S}_{0}^{4}(out)\end{array}]=[\begin{array}{cccc}{M}_{11}^{1} & {M}_{12}^{1} & {M}_{13}^{1} & {M}_{14}^{1}\\ {M}_{11}^{2} & {M}_{12}^{2} & {M}_{13}^{2} & {M}_{14}^{2}\\ {M}_{11}^{3} & {M}_{12}^{3} & {M}_{13}^{3} & {M}_{14}^{3}\\ {M}_{11}^{4} & {M}_{12}^{4} & {M}_{13}^{4} & {M}_{14}^{4}\end{array}][\begin{array}{c}{S}_{0}(in)\\ {S}_{1}(in)\\ {S}_{2}(in)\\ {S}_{3}(in)\end{array}]={M}_{\det }\cdot S(in)$$

In the experiments, light intensities of four measurements were recorded and used to calculate the input polarization with Eq. (3). Because the self-organized grating array and a polarizer were employed in our setup, *M*_det_ contains two parts:

4 $${M}_{\det }={M}_{polarizer}\cdot {M}_{grating}$$

Only zero-order diffraction exists in the transmitted light of the grating due to its sub-wavelength structure, a single grating can be modeled by a wave-plate with phase delay Δ and optical axis angle of *θ*:

5 $${M}_{grating}({\rm{\Delta }},\theta )=[\begin{array}{cccc}1 & 0 & 0 & 0\\ 0 & 1-(1-\,\cos \,{\rm{\Delta }}){\sin }^{2}\,2\theta  & (1-\,\cos \,{\rm{\Delta }})\sin \,2\theta \,\cos \,2\theta  & -\sin \,{\rm{\Delta }}\,\sin \,2\theta \\ 0 & (1-\,\cos \,{\rm{\Delta }})\sin \,2\theta \,\cos \,2\theta  & 1-(1-\,\cos \,{\rm{\Delta }}){\cos }^{2}2\theta  & \sin \,{\rm{\Delta }}\,\cos \,2\theta \\ 0 & \sin \,{\rm{\Delta }}\,\sin \,2\theta  & -\sin \,{\rm{\Delta }}\,\cos \,2\theta  & \cos \,{\rm{\Delta }}\end{array}]$$

The Müller matrix for the polarizer with optical axis angle of *φ* can be given by:

6 $${M}_{polarizor}(\phi )=\frac{1}{2}[\begin{array}{cccc}1 & \cos \,2\phi  & \sin \,2\phi  & 0\\ \cos \,2\phi  & {\cos }^{2}2\phi  & \sin \,2\phi \,\cos \,2\phi  & 0\\ \sin \,2\phi  & \sin \,2\phi \,\cos \,2\phi  & {\sin }^{2}2\phi  & 0\\ 0 & 0 & 0 & 0\end{array}]$$

The necessary condition for the existence of solution for Eq. (3) is that the determinant of *M*_det_ is nonzero. This condition can also be used to estimate the independence of four measurements. If $|{M}_{\det }|\ne 0$, the solution of Eq. (3) is:

7 $$S(in)={M}_{\det }^{-1}\cdot {S}_{0}^{i}(out)=\frac{{M}_{\det }^{\ast }}{|{M}_{\det }|}\cdot {S}_{0}^{i}(out)$$

## Experimental results and Discussion

**Figure 1:**
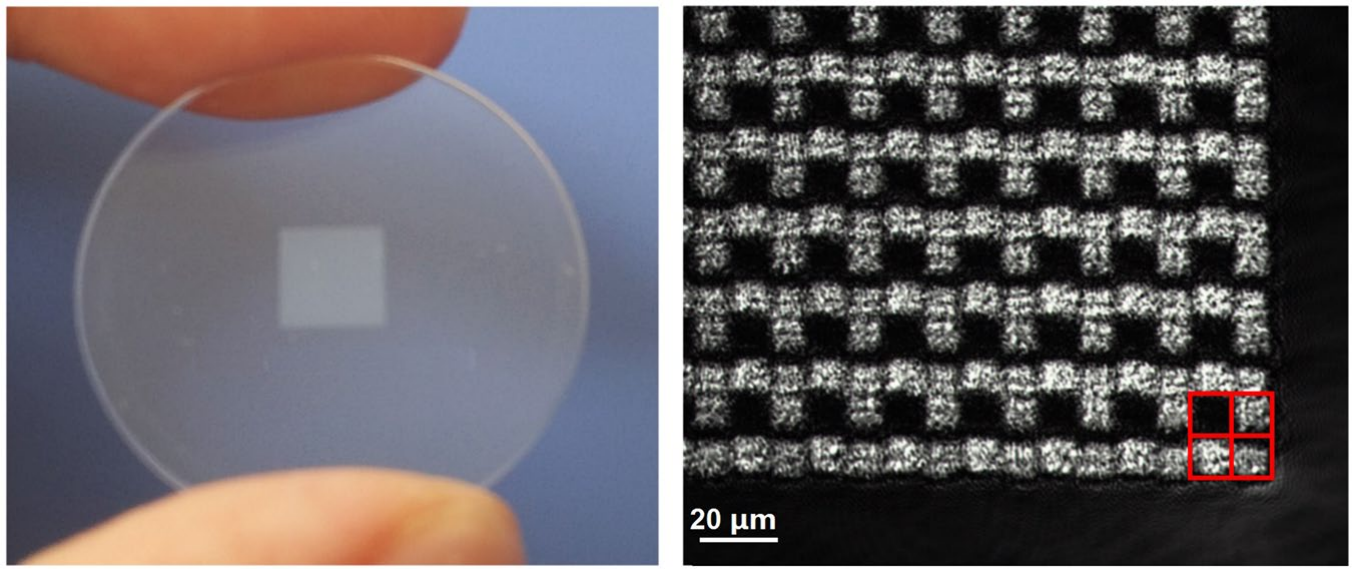
The grating array used in the experiments. (**a**) The grating array in a substrate of fused silica, (**b**) the micrograph of the grating array.

The self-organized grating array can be generated by modifying the optical refractive index when the ultra-fast laser tightly focuses into a fused silica with appropriate parameters. The typical energy is several sub-microjoule and the pulse duration is shorter than 200 fs. Theoretically, the period of the self-organized grating depends on the wavelength*λ*of the ultra-fast laser roughly described as T = λ/2n^25^. Here *n* is the refractive index of the material. For a Ti:sapphire laser with 800 nm wavelength, the grating period is about 267 nm, a wavelength much smaller than the visible and infrared light. Only zero-order diffraction of the transmitted light exists and a phase delay is induced by the strong birefringence of such gratings. The phase delay depends mainly on the modification of the material refractive index and thickness of the grating. Therefore, it can be controlled by adjusting the laser energy, pulse duration, and repetition rate, etc. Meanwhile, the direction of grating arrangement is perpendicular to the polarization of the ultra-fast laser. Thus, a two-dimensional wave-plate array is designable based on the self-organized gratings. The grating array used in our experiments is shown in Fig. 1(a). The single grating has the size of 10 × 10 microns. Altogether, 500*500 gratings (gray area in Fig. 1a) are tightly arranged in a fused silica substrate of 25.4 mm in diameter and 0.5 mm in thickness. The micrograph of the grating array between a pair of polarizers with orthogonal optical axes arrangement shows the clear rectangular gratings in Fig. 1(b). Each four adjoining gratings form a group for the detection of one Stokes vector. In an integrated polarimeter, the grating array is mounted close to the pixels of the detector. Because every grating has the same size as a single pixel on the image sensor, the DoFP polarimeter has a quarter resolution of the whole detector.

**Figure 2:**
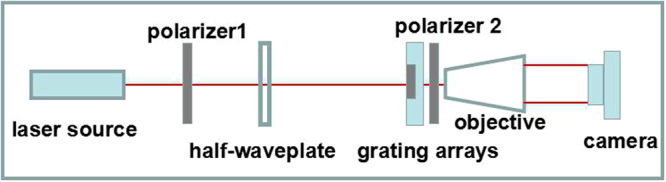
The experimental setup for the calibration of the grating array.

For the calibration of the grating array, a simple experimental setup was built as shown in Fig. 2. The polarization of the He-Ne laser at 632.8 nm was controlled by the first polarizer and a half-wave plate at the same wavelength. Then the light passes through the grating array and the second polarizer, transferring the polarization information to the intensity pattern. A 40x objective mirror was mounted just behind the second polarizer to magnify the intensity pattern recorded by a camera.

**Figure 3:**
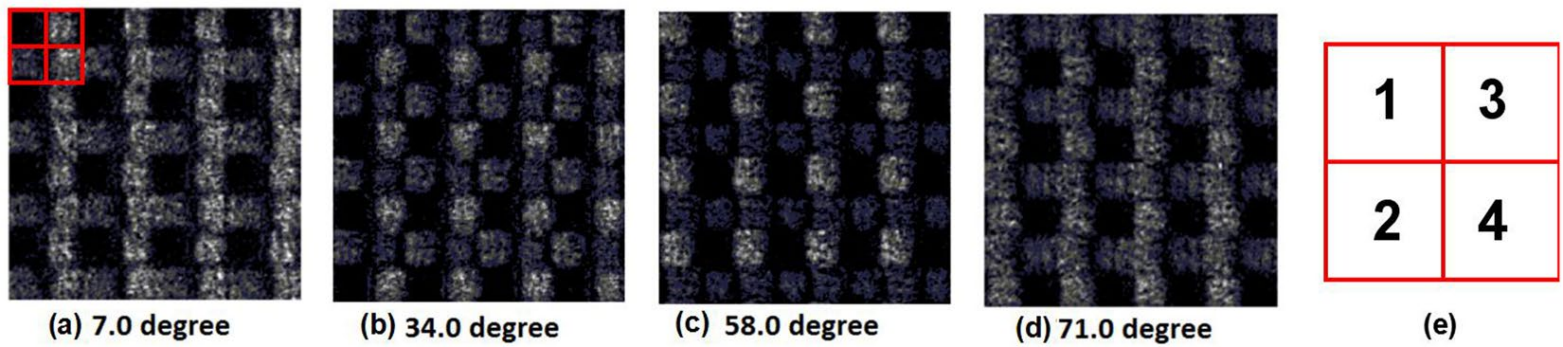
Measurements of grating axes by rotating the grating array between two polarizers with orthogonal optical axes.

Two experiments were implemented to acquire the optical axis direction and the phase delay of each single grating. In the first experiment, the angle-dependent transmissive image of the grating array between crossed polarizers was measured. The grating array was rotated around the direction of light propagation, and once the polarization of the input light is parallel or perpendicular to the axis of the single grating, no transmitted light was observed. The experimental results were shown in Fig. 3. The periodic grating elements were marked by a red frame and labeled from 1 to 4 as shown in Fig. 3(e). Grating axis directions are 7.0, 34.0,58.0 and 71.0 degrees respectively corresponding to Grating 1–4. The uncertainty of the angles is ±0.5 degree for a manual rotation operation. Different from the typical axis directions of 0, 45, 90, 135 degrees used in the partial Stokes vector measurement, the angles of 0, 30, 45 and 60 degrees of the gratings was designed to satisfy the nonzero of |*M*_det_|. However, the deviation of the manufactured grating from the designed values is perhaps due to the polarization angle deviation of the processing laser in a tight focusing condition.

Because no phase delay information can be obtained by simply rotating grating array, the polarization of the input light was rotated by the half-wave plate in place of the grating array in the second experiment. The light polarization was kept in the horizontal direction after the first polarizer. The Stokes vector of the light after a half-wave plate with the optical axis at angle *β* can be calculated as:

8 $$S(in)=[\begin{array}{cccc}1 & 0 & 0 & 0\\ 0 & \cos \,4\beta  & \sin \,4\beta  & 0\\ 0 & \sin \,4\beta  & -\cos \,4\beta  & 0\\ 0 & 0 & 0 & -1\end{array}]\cdot [\begin{array}{c}1\\ 1\\ 0\\ 0\end{array}]$$

The optical axis direction *φ* of the second polarizer was set at 90 degrees. After combining Eqs (3–6) and Eq. (8), the light intensity on the camera for each single grating can be calculated as:

9 $$I=({\sin }^{2}\,2\beta {\cos }^{2}{{\rm{\Delta }}}_{i}/2+{\sin }^{2}\,(2{\theta }_{i}-2\beta ){\sin }^{2}\,{{\rm{\Delta }}}_{i}/2)\cdot {T}_{i}$$

**Figure 4:**
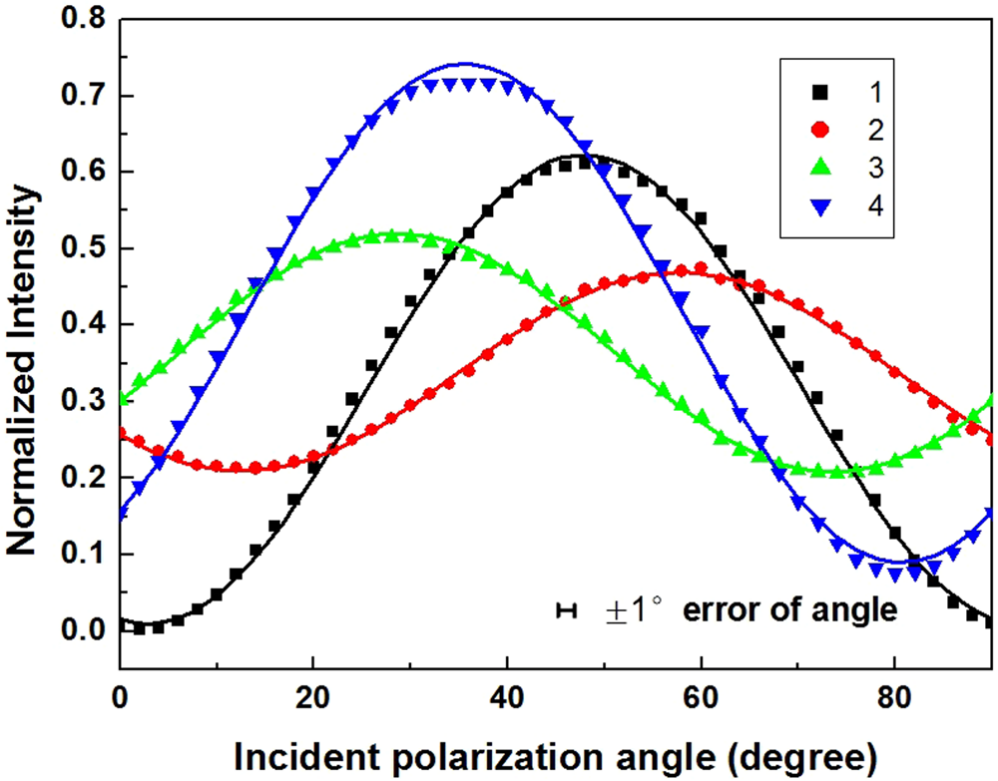
The measurement and data fit for the axes and the phase delay of four independent gratings. The dots are the experimental results for four gratings, and the curves are the simulation with Eq. (9).

In Eq. (9), *Δ* and *θ* are the phase delay and optical angle induced by the single grating. And the subscript represents the grating number as shown in Fig. 3(e). A new parameter *T* was utilized to simulate the polarization-independent transmissivity for each single grating. The experimental results and the simulation were plotted in Fig. 4. The dots represent experimental results for the four gratings, and the curves are the simulation results with Eq. (9). The extinction ratio of the combination of the nanograting and linear polarizer was calculated with the black curve 1 in Fig. 4, which denotes the grating with an optical axis close to the horizontal direction (7.43 degrees). After subtracting the background light intensity, the extinction ratio calculated with curve 1 is about 300. The signal to noise ratio was estimated with the light intensity divided by the background. A minimum value of 1.04 was achieved using the data in Fig. 4. The good agreements indicate that the self-organized grating can be properly described by a wave-plate model.

The grating parameters in Table 1 were produced by a nonlinear fitting based on Eq. (9). The axis angles agree well with the results in Fig. 3 within the range of errors. However, phase delays of the gratings are slightly inconsistent with each other. This may come from the process of manufacturing. The grating is distinguished by the different axis angle, which requires to change the polarization angle of the ultra-fast processing laser. The focusing condition will be slightly changed and results in the inhomogeneity of the grating phase delay. The same inhomogeneity exists in the transmissivity of the gratings. All the gratings need to be calibrated in theory. But considering the same machining process and good repeatability of the transmitted light intensity. We used the parameters on Table 1 for all the gratings on our sample.

**Table 1:** The fitted grating parameters with Eq. (8).

Gratings	Axis angle θ	Phase delayΔ	Transmissivity T
1	7.43 ± 0.4%	78.45 ± 0.8%	0.633 ± 0.5%
2	34.37 ± 0.3%	82.58 ± 0.2%	0.679 ± 0.2%
3	57.63 ± 0.2%	90.51 ± 0.2%	0.729 ± 0.2%
4	70.84 ± 0.5%	89.04 ± 1.1%	0.832 ± 0.5%

The Müller matrix for the grating array can be calculated from the data in Table 1. Scattering losses were ranged from 16.8% to 36.7% for the nanogratings in our experiments. They are attributed to the microscopic inhomogeneities and induced defect absorption. Because the subsequent linear polarizer largely blocks the scattering light, the quality of transmitted light intensity pattern can be preserved with presence of the scattering loss. When combined with the Müller matrix of a polarizer with axis direction at 90 degrees, Eq. (3) can be rewritten as:

10 $${M}_{\det }=[\begin{array}{cccc}0.500 & -0.110 & -0.235 & 0.441\\ 0.500 & -0.125 & 0.197 & 0.442\\ 0.500 & -0.493 & -0.082 & 0.000\\ 0.500 & -0.233 & 0.039 & 0.441\end{array}]$$

**Figure 5:**
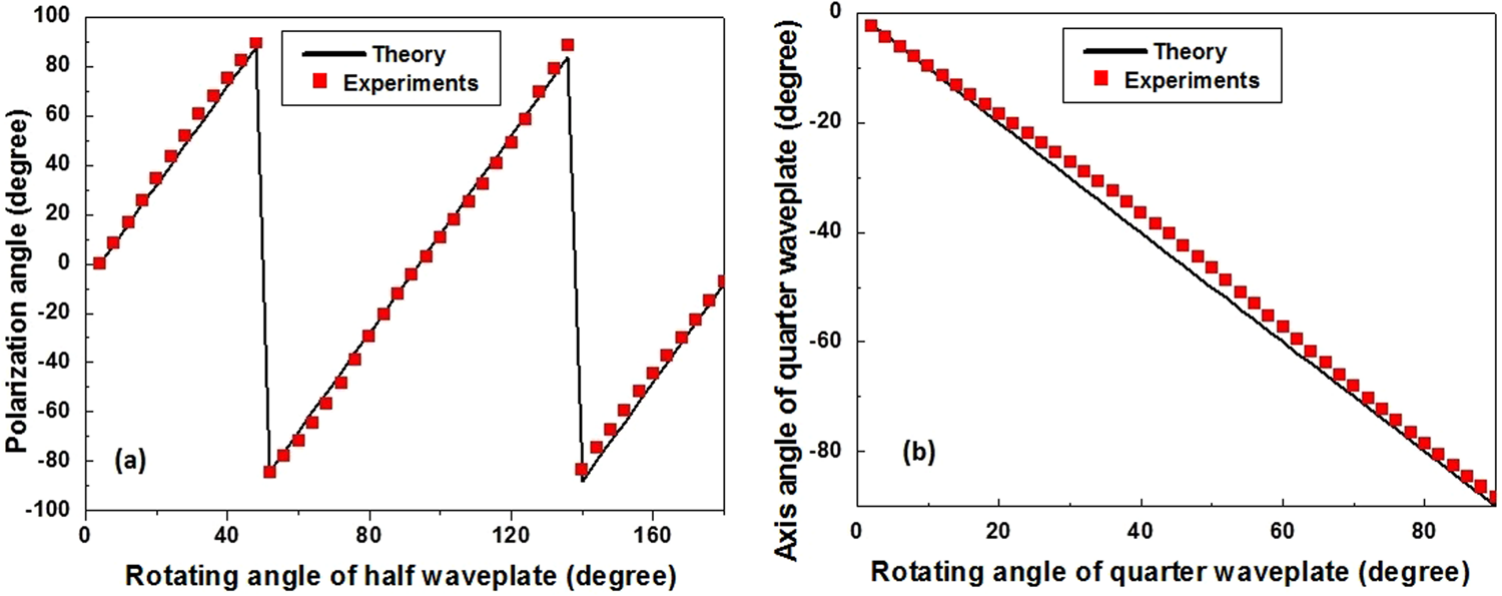
Measurements of linear (**a**) and elliptical (**b**) polarization generated by rotating a half waveplate and a quarter waveplate before a linear polarized light.

The measurements of linear and elliptical polarization generated by rotating a half waveplate and a quarter waveplate before a 0 degree linear polarized light were presented in Fig. 5. The linear polarization angle and axis angle of quarter waveplate were retrieved with Eqs (7) and (10) based on the experimental results. An uncertainty of ±1 degree for linear polarization angle and ±0.5 degrees for axis angle of quarter waveplate were estimated at a rotating manual operation, respectively. The deviation of the experimental results from the simulation is smaller than 5 degrees in both experiments. Especially, maximum deviation in Fig. 5(b) exists in the polarization with the quarter waveplate at 45 degrees. The results indicate there is a smaller error of the linear polarization measurement than the circular polarization in our setup. The residual deviation is attributed to cross-talk between different nanogratings and the deviation of the fabricated nanograting from a perfect phase delayer.

**Figure 6:**
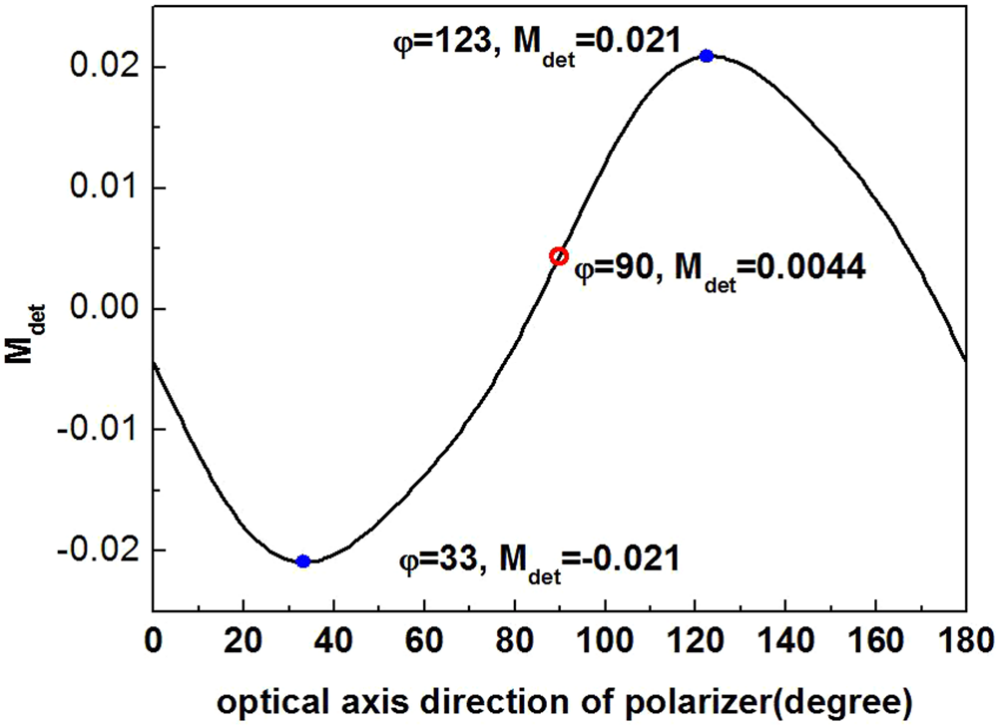
The optimization of the optical axis direction of the polarizer to obtain the minimum retrieval errors.

In Eq. (7), the retrieval Stokes vector is inversely proportional to the determinant of M_det_. Therefore the inversion error is smaller with a larger determinant of M_det_. However, |*M*_det_| = 0.0044 in Eq. (10), which is much smaller than the value of 0.2 in the ideal combination of polarization elements calculated by Sabatke *et al*.^28^. Because the phase delay and the optical axis of the gratings are fitted after the ultra-fast laser machining, the only optimization can be implemented with our sample is the adjustment of the axis direction of the subsequent polarizer, i.e., the angle *φ* in Eq. (6). The calculation in Fig. 6 shows that the optimum axis direction of the polarizer is 33 or 123 degrees with a maximum determinant of 0.021. That means 5 times error improvement by simply adjusting the polarizer angle.

**Figure 7:**
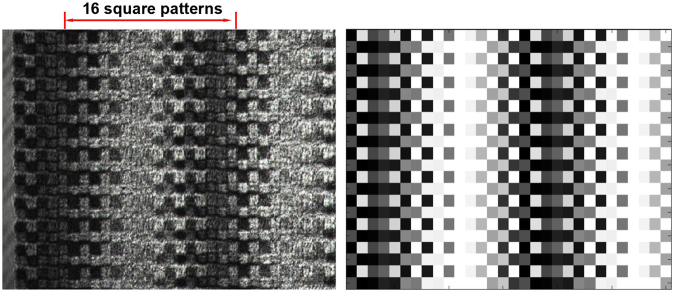
The experimental (**a**) and simulated (**b**) intensity patterns of the linear polarized light passing through a crystal wedge.

As a prototype verification, a linear polarized light at 45 degrees passing through a crystal wedge was measured with our setup. The wedge is made of calcite with refractive indexes of *n*_*e*_ = 1.4849 and *n*_*o*_ = 1.6557^29^. Optical fast and slow axes are at 0 degree and 90 degrees, respectively. The wedge angle *θ* is 1.3 ±0.2 degree along the horizontal direction, which induces a periodical phase delay at the x-direction to the input light. In order to obtain the best contrast ratio of the transmitted intensity patterns, the subsequent polarizer angle of −45 degrees was used to replace 33, 90 or 123 degrees. As a result, after the grating array and a polarizer, the light intensity was transferred to the periodical square patterns on the camera. The experimental result was shown in Fig. 7(a). The gray area on the left of Fig. 7(a) is the image of the grating edge. It was used to adjust the grating array on the focus position of the microscope objective. The intensity modulation period can be calculated as:

11 $$l=\frac{{\lambda }_{He-Ne}}{[({n}_{e}-{n}_{o})\cdot \,\tan \,\theta ]}=163.3\,\mu m$$

In Fig. 7(a), as we marked, about 16 square patterns comprise a light intensity period. It agrees with the size of 10 × 10 μm of a single grating. The Müller matrix of the crystal wedge can be calculated with Eq. (5) using the parameters of Δ(*x*) = *α* · *x* and *θ* = 0°. Here *α* is the linear coefficient of the phase delay induced by the crystal wedge. The simulated intensity pattern using Eqs (10) and (6) with *φ* = −45° was shown in Fig. 7(a). The standard deviation between experiment and simulation was calculated with Eq. (12):

12 $${\rm{Error}}=\frac{\sum _{\mathrm{i,j}}{[{I}_{\exp }(i,j)-{I}_{sim}(i,j)]}^{2}}{\sum _{\mathrm{i,j}}{I}_{sim}{(i,j)}^{2}}$$

Here *I*_*exp*_ and *I*_*sim*_ are the experimental and simulated light intensities, respectively. The intensity corresponding to single grating in Fig. 7(a) was averaged and marked with i and j to denote the x and y coordinates. The total error of 6.8% was calculated within 22 × 30 gratings in Fig. 7. Except for the major difference originated from the lower contrast ratio, the experimental result has a good agreement with the simulation. The error mainly comes from the inhomogeneous background and the light scattering.

## Conclusions

In this paper, a full-Stokes polarization imaging method based on a self-organized grating array was presented. The grating array was manufactured by the ultra-fast laser and constructed a two-dimensional phase modulator to manipulate the light polarization in the focal plane of the imaging detector. The calibration of the single grating can be theoretically completed with a wave-plate. The Müller matrix of the grating array was acquired and employed to optimize the measurement error. In the verification experiment, the intensity patterns of the light passing through a crystal wedge was measured and simulated. The consistency between the theory and the experiments shows that our prototype setup is capable of measuring the full-Stokes polarization in a microscopic field. Further improvements include integrating the grating array on top of pixels of the image sensor to achieve the compact configuration, increasing the resolution to beyond 500 × 500 by using a smaller grating array, optimizing the grating array to obtain four measurements which form a regular tetrahedron inscribed in the Poincaré Sphere. Combining the long-term stability, compact structure and simple manufacturing process, the self-organized grating array in fused silica is a promising approach for the development of full-Stokes DoFP polarimeter.
